# Community Empowerment and Community Partnerships in Nursing Decision-Making

**DOI:** 10.3390/healthcare7020076

**Published:** 2019-06-12

**Authors:** Pedro Melo, Odete Alves

**Affiliations:** 1 Centre for Interdisciplinary Health Research/School of Nursing, Universidade Católica Portuguesa, 4169-005 Porto, Portugal; 2 Institute of Biomedical Sciences Abel Salazar (ICBAS), Universidade do Porto, 4050-313 Porto, Portugal; odetemaalves@gmail.com

**Keywords:** Community health nursing, community empowerment, community partnerships, decision-making

## Abstract

Community empowerment has been studied as a process and result phenomenon throughout the last 40 years. Community partnership, which has been studied during the last 20 years, has been identified as a key process to promote intervention and research within communities. In this paper, we introduce the relation between these two concepts, from the research that is being developed at the Centre for Interdisciplinary Health Research (CIIS) in Universidade Católica Portuguesa. We comment on the available evidence regarding community partnership and community empowerment within the Nursing Decision-Making process. There is a particular focus on Community Health Nursing Specialists (CHNS) and the aim to promote the identification of CHNS as potential community partnership developers within society. It is also important to analyze how community partnership processes are intentionally integrated as a nursing intervention within the nursing process. This analysis should occur from the nursing diagnosis to the evaluation of health gains in communities sensitive to CHNS care in a Nursing Theoretical Model developed from a Nursing PhD process—the Community Assessment, Intervention, and Empowerment Model.

## 1. Introduction

Community Health Nursing (CHN) in Portugal has its central skills published in law [1] and organized into four main skill categories: epidemiological survey, health planning, program and project management, and groups and community empowerment. 

The Community Assessment, Intervention, and Empowerment Model (MAIEC) [2,3] is a nursing theoretical model that bases clinical decision-making in CHN care on approaching communities as a care unit of nurses. This model presents the main definitions of community, community environment, community health, and community nursing care, which are the assumptions and theoretical postulates that sustain collaborative decision-making between the nurse and the community [2,3,4,5].

The MAIEC, based on the nursing decision-making process proposed by Figueiredo [6], presents a decision-making matrix for a community as a nursing client based on community empowerment as a process and a result of nursing care. This matrix includes information about nursing’s focus (community management) and diagnostic dimensions, and the nursing foci (community participation, community process, and community leadership) [2] that are included in the International Classification on Nursing Practice (ICNP) [7]. Nursing interventions are described for the different possible diagnoses. One of them is “to promote partnerships”.

The aim of this paper is to introduce the research process that we are developing at the Nursing Research Platform at the Centre for Interdisciplinary Health Research (CIIS) at Universidade Católica Portuguesa. Specifically, we consider our hypothesis that the intervention “to promote partnerships” has a relationship with community empowerment development, particularly with community partnerships. We started with the questions: How are community empowerment and community partnerships related to Community Health Nursing? How do community health nurses integrate the community partnerships process in their decision-making?

## 2. Community Empowerment and Community Health Nursing

The number of studies on community empowerment has significantly increased over the last 30 years [8,9,10,11,12,13]. This can be seen through the empowerment of neighborhood communities in the United States that have social problems, such as poverty, drug addiction, or violence. Various studies have continued to relate it to other health problems, specifically in relation to public health. Laverack proposes an assessment of community empowerment based on nine domains [13]. The “community participation” domain involves the active participation of community members in solving their own community problems. The “problem assessment capacities” domain is related to community skills to identify problems, solutions, and actions to solve those problems. The “local leadership” domain integrates the support of a leader into the community. The “organizational structure” domain identifies the existence of structures, such as committees, that are concerned with solutions to community problems. The “resource mobilization” domain considers the existence and raising of resources to offer solutions to community problems. “Links to others” relates to the connection of a community to other communities or sources of resources. The “ability to ask why” is an indicator of a community’s ability to self-analyze its own condition. “Program management” refers to the capacity of the community to manage its intervention programs independently of external agents. “The relationship with outside agents” is the last community empowerment domain, which considers the external agent facilitation alongside the autonomy of community to solve its problems. These nine domains constitute an important instrument to assess community empowerment, called the “Empowerment Assessment Rating Scale” [13]. Alongside this assessment of community empowerment, Laverack explains the process of community empowerment using the Continuous Model of Community Empowerment, which was developed in different stages. This model starts with personal action (related to individual empowerment) and progresses to social and political action [14].

**Figure 1:**
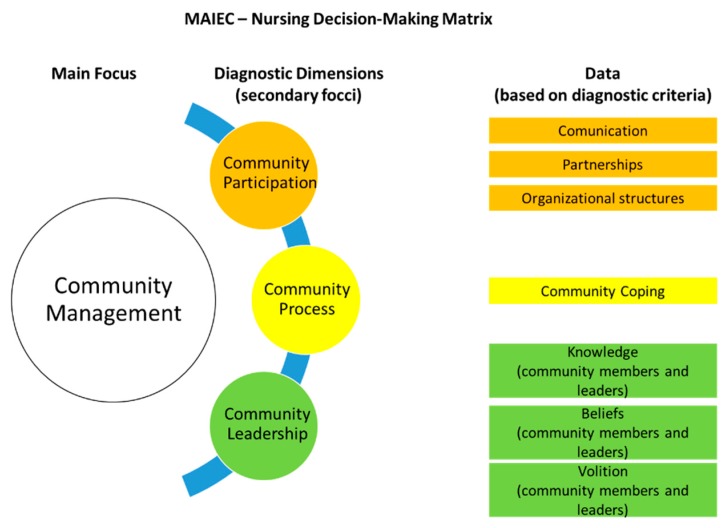
The Community Assessment, Intervention, and Empowerment Model (MAIEC)’s nursing diagnosis matrix for a community.

There is a lack of studies on community empowerment in Community Health Nursing. In 2005, Falk-Rafael identified in her study that nurses could promote community empowerment in their practice at the intersection of public policy and personal lives [15]. In addition, a recent study has suggested that community health nurses contribute to the empowerment of Afro-Colombian community leaders by promoting control and social participation according to self-knowledge, organization, community engagement, intersectional work, and expanding project management [16]. In 2013, a report from an Irish Population Health interest group identified community empowerment as an intervention for public health nurses, more specifically concerning the building of coalitions between health professionals and community groups to solve specific community issues, such as improvement in breastfeeding rates [17]. Within the Portuguese political context and national legislation are four main community health nursing skills, one of which is community empowerment. Community empowerment is associated with an approach to CHN in which the community is the unit of nursing care. However, when we analyze the existing conceptual models and theories of nursing, we find that, in the majority of these studies, the community exists as a context of care to individuals and/or families [6,18]. The MAIEC [2,3,4] is a conceptual framework and prescriptive theory that defines the concept of community as a set of people in a defined geographical context with functions that have been identified for its members and for the groups and organizations that they form. It requires a sense of identity that allows for the sharing of common goals. It is also an open system that results from the interaction of individuals, groups, and community organizations. The whole and the parts interact through community participation, leadership, and processes [4]. The MAIEC integrates community empowerment within the definition of community health, describing it as a dynamic process of community empowerment in which community participation, processes, and leadership combine in order to be able to identify and solve problems and mobilize community resources. Community health determinants are related to public health determinants in terms of community empowerment indicators, and enhance the effectiveness of a community intervention from a collaborative perspective [2,4]. In this way, nursing care to the community as a unit is defined by the MAIEC as the development of a collaborative and empowering action by the community that grounds the approach of community management as an enhancer of community health gains sensitive to nursing care. It conceptualizes the community empowerment approach as a process in an integrated and integrative view of the community as a whole and that results in the community’s achieving the highest level of community empowerment [2,3,4]. This model also has a decision-making matrix [2,4], which is based on the ICNP [7], that places a central focus on nursing attention (community management) and three diagnostic dimensions (community participation, community process, and community leadership). Each dimension has specific diagnostic criteria whose satisfaction may lead to a judgment that forms a main nursing diagnosis (e.g., community management commitment) and a secondary diagnosis (e.g., community leadership commitment: knowledge of leaders not demonstrated. The MAIEC was developed from a constructivist perspective using two research techniques: the focus group, which was comprised of Portuguese community health nurses, and the Delphi panel, which was comprised of a population of community health nurses from Portugal [2,3]. Figure 1 shows an illustration of the relation between the nursing focus and data in the MAIEC decision-making matrix. 

Considering that the MAIEC functions as a diagnostic decision-making for nursing care to the community as client, community partnerships appear to be associated with the secondary focus of community participation that is related to the interaction of the community with both the internal system and with other community subsystems [2,3].

## 3. Community Partnerships and Community Empowerment

Community partnerships are analyzed as a strategy, integrated into community-based interventions (CBIs), and have been deeply studied over the last two decades [19,20,21,22]. In the context of CBIs, partnerships emerge from a continuous relation between an external agent and community members and organizations for the purpose of mobilizing resources and knowledge and promoting community intervention projects. Researchers also identify community partnerships as a process that improves communication between organizations and people inside communities to make collective goals clearer and more commonly understood [23] or to develop new resources for individuals to access and use to solve problems, such as the provision of support to Diabetic Prevention Services in youth [24]. Other researchers include community partnerships within the process of Community-Based Participatory Research (CBPR) [25], and consider the trust between a member of a community and external agents that develop research to be the key to a community partnership in CBPR.

Regarding community empowerment, partnerships are identified as a higher stage of the community empowerment process [14], which is attained just before the acquisition of the level of political and social action. According to CHN’s community empowerment approach, the MAIEC describes partnerships as data that serve as diagnostic criteria for the community participation focus, integrated in the main diagnosis on the community management nursing focus, as shown in Figure 1. Following this diagnosis, the MAIEC suggests that, in a situation where community partnerships do not exist, which enables the sub-diagnosis of community participation being in jeopardy, one of the interventions might be “to promote community partnerships”.

Community involvement in genuine community partnerships is increasingly being accepted as best practice in community intervention projects, despite the many challenges, barriers, obstacles, and difficulties in doing so [26].

The process of promoting community partnerships implies, at an early stage, the identification of potential partners in the community who are interested in sharing a common purpose. Together, stakeholders should question why the partnership is necessary to achieve the purpose. The recognition of the problems and goals to be achieved is the basis of this first contact [27].

Use of the partnership life-cycle model, which represents the status of a partnership, could help its members to understand where they are, why they are there, and the conditions that need to be satisfied and then maintained to realize the full potential of the partnership [27].

**Figure 2:**
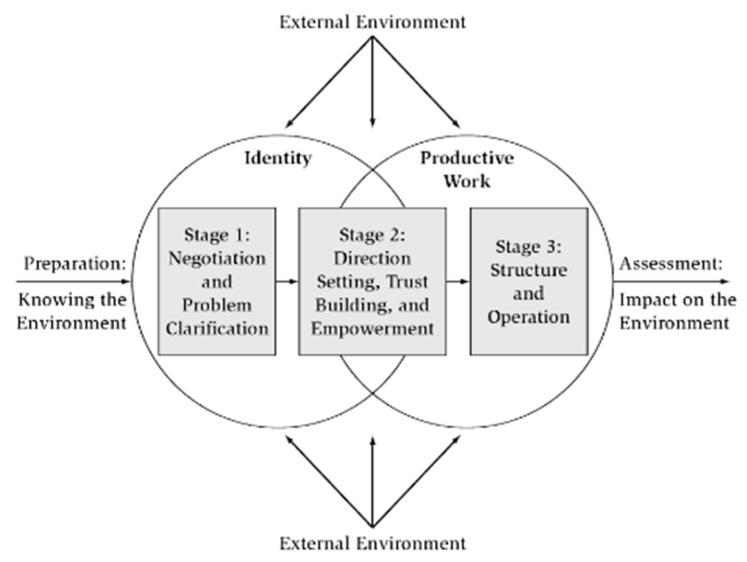
Framework for Partnerships for Community Development [28].

Habana-Hafner, Reed, and Associates (1989) developed the framework for Partnerships for Community Development for Organizational and Community Development (University of Massachusetts, Amherst) [28]. This framework, which is presented in Figure 2, has two abstract concepts at its essence: (1) identity—the membership in a group, its shared sense of meaning and goals, values, and culture, interpersonal relations, shared resources, history, and synergy; and (2) productive work—leadership, decision-making, communication, policies and rules, roles, evaluation, organizational structure, and group dynamics. The partnership must interact with a dynamic and persistent external environment with such influences as geography, history, politics, economics, power structure, and ethnic and cultural diversity. Three stages of development occur as organizations work together in the partnership. Prior to Stage 1 is preparation: Knowing the environment, which involves clarifying broad concerns, exploring environmental influences, and defining membership. Stage 1 is Negotiation and Problem Clarification; Stage 2 is Direction Setting, Trust Building, and Empowerment; and Stage 3 is Developing a Structure and Operations. Following Stage 3 is Assessment: Impact on the Environment, in which the partnership evaluates its outcomes. The partnership may then end, depending on different strategies to overcome the initial problem, or emphasis on another problem [29].

For the maintenance and sustainability of a partnership, it is important to know, through joint reflection, the current level of collaboration between members and whether collaborative efforts have maximized the effectiveness of the partner organization [29]. Various authors state that the sustainability of a community partnership is anchored in continued training and technical assistance to build upon the partnership’s capacity and influence [29,30,31,32,33,34,35,36].

In addition, to sustain collaboration, resources and strategic program planning must also exist. Planning must be both short-term and long-term. The collaboration must be able to identify emerging trends and issues and develop strategies for any necessary expansion [36].

The active and democratic participation of a community in community development results in community empowerment, which, according to the World Health Organization (WHO, 2009), implies not only the involvement and participation of communities in their own development, but also community ownership and action that explicitly aims at social and political change [35]. These are essential conditions that partnerships must satisfy for interventions to be successful, with a view to working towards sustainable community development [35,36,37,38]. Community development cannot occur if citizens do not participate in projects/programmes [37].

Coalition building promotes and develops alliances among organizations or constituencies for a common purpose. It builds linkages, solves problems, and/or enhances local leadership to address health concerns [38,39].

Regarding community health nursing, a recent study identified community partnerships as important to community resilience in vulnerable communities [40]. Public health nurses use strategies to promote stakeholder involvement, such as forums and education sessions to increase a community’s awareness of its strengths, and promote a community’s capability to engage all stakeholders to bring their skills together to promote community partnerships to respond to vulnerability in situations such as natural catastrophes.

## 4. Discussion and Conclusions

Considering the framework analyzed above, we found a clear relationship between nursing decision-making related to a community as a care unit of nurses and community empowerment, integrating community partnerships into nursing diagnostic criteria and interventions. In this context, a community partnership is a complex process of leadership construction in communities and is enforced by the diagnostic dimension community participation in the MAIEC Decision-Making matrix [2]. The framework for Partnerships for Community Development [25] is a valuable reference for the assessment and promotion of community partnerships.

Nursing interventions, such as “negotiate partnership”, “advocate partnership”, “promote partnership”, and “manage partnership” are clearly related to this framework, and are included in the role of community health nurses in promoting community partnerships, in a systemic and multidisciplinary approach to community health, for the empowerment of a community both as a process and as a result.

Using theoretical models, such as the MAIEC, community health nurses are able to base the nursing profession on the nursing discipline, introduce the community as a client into the nursing process, make community health nursing diagnoses, and prescribe nursing interventions to respond to those diagnoses.

Interventions related to community participation, which include community partnerships, allow for the promotion of community empowerment as a process of nursing decision-making. Nursing interventions also allow for the promotion of efficient community management to make a community the “owner” of its own problems, which is an approach that has been upheld by various researchers since the 1980s and is currently upheld within the modern research context [8,9,10,11,12,13,14].

Although there exist difficulties, barriers, and obstacles in the promotion of community partnerships [23], community health nurses have, in an organizational context, proximity to communities. This fact facilitates their active participation in the promotion of cohesion and the construction of common objectives. Nurses are, in this way, an important link in a multidisciplinary team whose objective is to promote effective community partnerships based on community empowerment.

It is necessary to carry out more studies to identify specific models of promotion of community partnerships, integrated in the nursing process, that allow for the identification of nursing diagnoses and interventions that specifically bear upon community partnerships. However, the existence of a nursing model that identifies a community partnership based on diagnostic criteria based on community empowerment is an important step to achieve this, and represents a challenge to organizations that count on community health nurses to promote community partnerships and community empowerment related to community health.

At the CIIS, we are developing a quasi-experimental study with different kinds of communities to test the influence of the MAIEC on the development of community empowerment, implement the interventions suggested by the decision-making matrix of this Theoretical Nursing Model, and assess the health gains sensitive to nursing care of communities, including the improvement of community partnerships. We are also developing studies on the barriers to and facilitators of these community partnerships to contribute to the identification of CHNS contributions to community empowerment in general and to community partnerships specifically.
